# *MCPggaac* haplotype is associated with poor graft survival in kidney transplant recipients with *de novo* thrombotic microangiopathy

**DOI:** 10.3389/fimmu.2022.985766

**Published:** 2022-09-14

**Authors:** Vojtech Petr, Dorottya Csuka, Petra Hruba, Ágnes Szilágyi, Marek Kollar, Antonij Slavcev, Zoltán Prohászka, Ondrej Viklicky

**Affiliations:** ^1^ Department of Nephrology, Institute for Clinical and Experimental Medicine, Prague, Czechia; ^2^ First Faculty of Medicine, Charles University, Prague, Czechia; ^3^ Research Laboratory, Department of Internal Medicine and Hematology, Semmelweis University, Budapest, Hungary; ^4^ Transplant Laboratory, Institute for Clinical and Experimental Medicine, Prague, Czechia; ^5^ Department of Clinical and Transplant Pathology, Institute for Clinical and Experimental Medicine, Prague, Czechia; ^6^ Department of Immunogenetics, Institute for Clinical and Experimental Medicine, Prague, Czechia

**Keywords:** kidney transplantation, complement, haplotype, thrombotic microangiopathy, rejection

## Abstract

*De novo* thrombotic microangiopathy (TMA) is associated with poor kidney graft survival, and as we previously described, it is a recipient driven process with suspected genetic background. Direct Sanger sequencing was performed in 90 KTR with *de novo* TMA and 90 corresponding donors on selected regions in *CFH, CD46, C3*, and *CFB* genes that involve variations with a functional effect or confer a risk for aHUS. Additionally, 37 recipients of paired kidneys who did not develop TMA were analyzed for the *MCPggaac* haplotype. Three-years death-censored graft survival was assessed using Kaplan-Meier and Cox regression models. The distribution of haplotypes in all groups was in the Hardy-Weinberg equilibrium and there was no clustering of haplotypes in any group. In the TMA group, we found that *MCPggaac* haplotype carriers were at a significantly higher risk of graft loss compared to individuals with the wild-type genotype. Worse 3-year death-censored graft survival was associated with longer cold ischemia time (HR 1.20, 95% CI 1.06, 1.36) and recipients’ *MCPggaac* haplotype (HR 3.83, 95% CI 1.42, 10.4) in the multivariable Cox regression model. There was no association between donor haplotypes and kidney graft survival. Similarly, there was no effect of the *MCPggaac* haplotype on 3-year graft survival in recipients of paired kidneys without *de novo* TMA. Kidney transplant recipients carrying the *MCPggaac* haplotype with *de novo* TMA are at an increased risk of premature graft loss. These patients might benefit from therapeutic strategies based on complement inhibition.

## Introduction

*De novo* thrombotic microangiopathy (TMA) represents a rare complication of kidney transplantation associated with delayed graft function and impaired graft survival. The pathogenesis of *de novo* TMA remains poorly understood. We have previously shown that *de novo* TMA is primarily recipient-driven and that rejection-associated TMA has the worst prognosis (1).

While the TMA in atypical hemolytic uremic syndrome frequently occurs as a result of inherited or autoimmune dysregulation of the complement system, complement gene alterations are now being increasingly described in secondary TMA (2). *De novo* TMA in kidney transplantation is usually considered to be secondary and associated with longer cold ischemia time, exposure to calcineurin inhibitors or antibody mediated rejection (1, 3). Several studies, however, point to a possibility of a hereditary background (4, 5). We hypothesized that disease modifying variants of complement genes may influence the development and the impacts of *de novo* TMA after kidney transplantation.

In order to elucidate a possible link between complement gene variants and *de novo* TMA, we analyzed single nucleotide polymorphisms (SNP) and haplotypes of selected genes known to be associated with aHUS or dense deposit disease for their association with *de novo* TMA in kidney transplant recipients (KTR) and their corresponding donors. Chosen SNPs and haplotypes include complement factor H (master regulator of alternative pathway), membrane cofactor protein (CD46, membrane bound cofactor for complement regulatory proteins), complement factor B (cofactor of alternative pathway activation), and C3 component (core component of all complement pathways).

## Methods

### Participants

**Figure 1:**
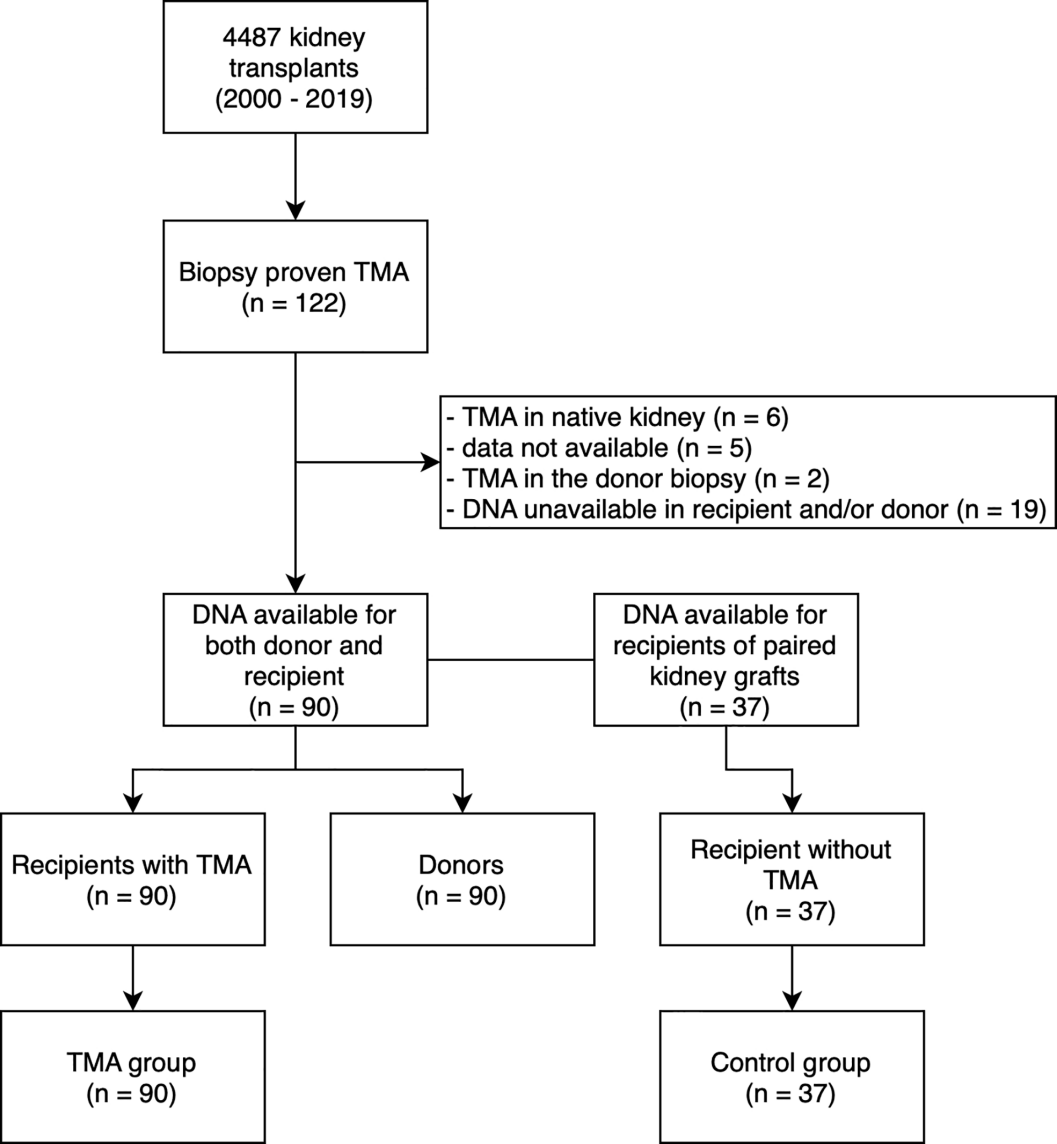
Study flow diagram. TMA, thrombotic microangiopathy; DNA, deoxyribonucleic acid.

This is a retrospective analysis of selected risk variants and haplotypes of complement genes in KTR with *de novo* TMA. We retrospectively evaluated histological reports of 4487 kidney transplant recipients from 2000 and 2019 and found 122 biopsies with histological patterns/signs of TMA, this cohort has been described previously (1). We excluded KTR with unavailable DNA samples, incomplete data sets, cases of recurrent TMA, donor-derived TMA, and living donor transplants. Finally, we enrolled 90 remaining KTR and identified donors of their kidney grafts, all donor DNA samples were available. The TMA group (n = 90) and the donor group (n = 90) were established (**Figure 1**).

In order to assess the interaction between TMA and the *MCPggaac* haplotype (see below), we have performed sequencing of *CD46* in recipients of paired kidney grafts who did not develop TMA (control group, n = 37) (**Figure 1**). Since the remaining paired kidneys were transplanted outside of our transplant center, additional DNA samples were unavailable. Clinical and survival data were collected from medical records and the transplant registry, respectively. The study was approved by the Institutional review board of the Institute for Clinical and Experimental Medicine, Prague, Czech Republic. The study is in accordance with the 1964 Helsinki Declaration and its later amendments. The clinical and research activities being reported are in accordance with the Principles of the Declaration of Istanbul as outlined in the “Declaration of Istanbul on Organ Trafficking and Transplant Tourism”.

### Genetic analysis

Genomic DNA was extracted from the recipients’ and donors’ peripheral lymphocytes for molecular genetic analysis. In order to determine the genotype of particular variations that have a functional effect or confer a risk for aHUS or dense deposit disease, selected regions of complement Factor H (*CFH*, promoter, exons 9, 14, 19), membrane cofactor protein (*CD46*, promoter, exon 10), Factor B (*CFB*, exon 2) and C3 (*C3*, exons 3, 9) were analyzed by PCR amplification followed by direct bidirectional DNA sequencing. After treatment with exonuclease I and alkaline phosphatase, PCR products were processed for sequencing applying BigDye v3.1 sequencing chemistry (Applied Biosystems, Foster City, CA) and sequenced using an ABI 3130xl Genetic Analyzer (Applied Biosystems). Sequencing chromatograms were visualized using CLC DNA Workbench 21 (CLC Bio, Aarhus, Denmark). Presence of the *CFH* H3 and *CD46 MCPggaac* risk haplotypes was deduced from genotype data of the variations identified in *CFH* and *CD46*. Those patients, who concurrently carried the T allele of −331C/T, C allele of c.1204C>T, G allele of c.2016A/G and T allele of c.2808G/T were regarded as heterozygote H3 carriers, while homozygotes were regarded as homozygote H3 carriers. Carrier state of the MCPggaac haplotype was similarly determined based on the genotype of three of its constituents (c.-652A/G, c.-366A/G and IVS9-78G/A) as the other two constituents (rs859705 (IVS12638A/G) and rs7144 (c.2232C/T) are strongly linked to the IVS9−78G/A variation (PMID: 23787556) (**Supplementary Table 1**). Primer sequences and PCR conditions are available upon request.

### Pathological definitions

TMA diagnosis was based on the presence of 1 or more fibrin thrombi in glomeruli or small arteries and arterioles; endothelial swelling with luminal compromise of glomerular capillaries with or without fragmented erythrocytes; and vascular fibrinoid necrosis or mucoid thickening of intima of small arteries/arterioles. The biopsies with TMA were reassessed by a pathologist (M. K.) for the purpose of this study. All biopsies were reassessed according to the most recent Banff 2019 classification (6). TMA with rejection was defined as a concomitant finding of TMA and rejection in the same biopsy. Selected Banff scores of TMA diagnosis biopsies are given in **Supplementary Table 2**.

### Statistics

The statistics were calculated using R-Studio software, version 1.2.5042 (Development for RStudio, Inc., Boston, MA). Continuous variables were reported as medians and interquartile ranges (IQRs), and the Mann-Whitney U test was used for a simple comparison of groups in univariable analysis. Categorical variables were reported as proportions and chi-square or Fisher exact tests were used as appropriate. The Kaplan-Meier method and log-rank test were used to analyze time to graft loss. The graft survival was analyzed from the time of TMA diagnosis, follow-up time of the corresponding paired kidney graft was counted from the same time-point. Event of interest was graft loss defined as return to dialysis or retransplantation. Patients were censored after 3 years or upon death with a functional graft. Cox proportional hazards model was used to estimate hazard ratios (HRs) and 95% CIs for kidney allograft loss. An overall P value <0.05 was considered statistically significant.

## Results

### Basic characteristics of the study groups

Clinical and demographical data are presented in **Table 1**. There were no significant differences between the TMA group and the control group. Median time from transplantation to TMA diagnosis was 9 days (IQR 6 – 42.5). All biopsies were performed for cause. List of causes of end stage kidney disease, hematologic manifestations of *de novo* TMA, the maintenance immunosuppression at the time of diagnosis of TMA, and the treatment of TMA are available in **Supplementary Tables 3–6**.

**Table 1:** Basic characteristics and demographics.

Parameter	TMA group (n = 90)	Control group (n = 37)	p-value
**Median recipient age, years (IQR)**	52 (43 – 59)	54 (47 – 62)	0.092
**Female recipient, No. (%)**	55 (61.1)	22 (59.5)	1
**Median donor age, years (IQR)**	55 (43 – 60)	56 (51 – 60)	0.258
**Female donor, No. (%)**	41 (46.1)	18 (48.6)	0.945
**Median cold ischemia time, hours (IQR)**	16.5 (14.3 – 18.9)	15.8 (12.3 – 19.8)	0.561
**Median dialysis vintage duration, months (IQR)**	22 (12.3 – 40)	26 (15 – 35)	0.371
**Peak preTx PRA, median (IQR)**	8 (2 – 30)	6 (2 – 18)	0.835
**HLA mismatch, median (IQR)**	3 (3 – 4)	3 (3 – 4.25)	0.728
**Retransplantation, No. (%)**	15 (16.7)	5 (13.5)	0.792
**T-cell depletive induction, No. (%)**	40 (44.4)	17 (45.9)	1

TMA, thrombotic microangiopathy; SD, standard deviation; IQR, interquartile range; preTx, pretransplantation; PRA, panel-reactive antibodies; HLA, human leukocyte antigen.

### Distribution of SNPs and haplotypes in recipients and donors

Selected single nucleotide polymorphisms and haplotypes of complement genes that are known to be associated with aHUS or dense deposit disease were analyzed. The distributions of SNPs and haplotypes in the TMA group, the control group and the donor group were similar to that of the general population. The haplotype distribution was within the Hardy-Weinberg equilibrium (**Table 2**).

**Table 2:** Single nucleotide polymorphisms and haplotypes in the TMA group, donors, and the control group.

Gene	SNP/haplotype (Reference SNP cluster ID)	Recipients with *de novo* TMA, n (%)			Recipients without *de novo* TMA, n (%)			Donors, n (%)		
		Wild-type homozygote	Heterozygote	Variant homozygote	Wild-type homozygote	Heterozygote	Variant homozygote	Wild-type homozygote	Heterozygote	Variant homozygote
***CFH***	-257C/T (rs3753394)	40 (48.8)	35 (42.7)	7 (8.5)				46 (51.7)	41 (46.1)	2 (2.2)
	Y402H (rs1061170)	29 (34.5)	38 (45.2)	17 (20.2)				33 (36.7)	40 (44.4)	17 (18.9)
	Q672Q (rs3753396)	59 (69.4)	23 (27.1)	3 (3.5)				60 (67.4)	26 (29.2)	3 (3.4)
	E936D (rs1065489)	58 (69)	23 (27.4)	3 (3.6)				63 (70)	23 (25.6)	4 (4.4)
	CFH H3 haplotype	63 (75)	18 (21.4)	3 (3.6)				65 (72.2)	24 (26.7)	1 (1.1)
***CD46***	c.-652A/G (rs2796267)	37 (43.5)	37 (43.5)	11 (12.9)	19 (51.4)	11 (29.7)	7 (18.9)	37 (41.6)	40 (44.9)	12 (13.5)
	c.-366A/G (rs2796268)	30 (35.3)	46 (54.1)	9 (10.6)	19 (51.4)	13 (35.1)	5 (13.5)	40 (44.9)	37 (41.6)	12 (13.5)
	IVS9-78G/A (rs1962149)	31 (36.9)	46 (54.8)	7 (8.3)	20 (54.1)	12 (32.4)	5 (13.5)	42 (47.2)	34 (38.2)	13 (14.6)
	*MCPggaac* haplotype	42 (49.4)	37 (43.5)	6 (7.1)	22 (59.5)	11 (29.7)	4 (10.8)	47 (52.8)	33 (37.1)	9 (10.1)
***C3***	R102G (rs2230199)	58 (69)	25 (29.8)	1 (1.2)				65 (72.2)	20 (22.2)	5 (5.6)
	P314L (rs1047286)	58 (69)	25 (29.8)	1 (1.2)				64 (71.1)	22 (24.4)	4 (4.4)
***CFB***	R32W (rs12614)	65 (77.4)	18 (21.4)	1 (1.2)				73 (81.1)	16 (17.8)	1 (1.1)
	R32Q (rs641153)	66 (78.6)	17 (20.2)	1 (1.2)				83 (92.2)	6 (6.7)	1 (1.1)
	CFB R32W/Q haplotypes	49 (58.3)	31 (36.9)	4 (4.8)				66 (73.3)	22 (24.4)	2 (2.2)

SNP, single nucleotide polymorphism; CFH, complement factor H; CD46, cluster of differentiation 46; C3, complement factor 3; CFB, complement factor B; WT HM, wild-type homozygote; HT, heterozygote; variant HM, variant homozygote; TMA, thrombotic microangiopathy.

Total number of subjects in case of each SNP might differ as the quality of DNA was not of sufficient for determining all loci in all recipients/donors.

### Associations with clinical outcomes

Next, the association between SNPs and haplotypes and death-censored graft was analyzed. Interestingly, the *MCPggaac* haplotype in recipients was associated with a significantly higher risk of graft loss in three years (**Supplementary Table 7**). There was also signal for similar association between H3 haplotype of CFH in donors, however, this result was based on one patient with primary non-function. Therefore, we decided not to focus on this finding further.

### Graft survival in recipients with TMA and in paired kidneys

Three-year death-censored graft survival was substantially lower in the TMA group compared to the control group (67.2% vs. 91.1%, **Supplementary Figure 1**). Furthermore, graft survival in the TMA group was significantly worse in those associated with ABMR (39.2%) compared to TCMR or rejection-free subgroups (72.7% and 73.2%, **Supplementary Figure 2**). The total follow-up was 403 person-years in the TMA group (median 3.6; maximum 17.2 y) and 258 person-years in the control group (median 6.1; maximum 17.2 y); no patient was lost to the follow-up.

### Graft survival and MCPggaac haplotype

**Figure 2:**
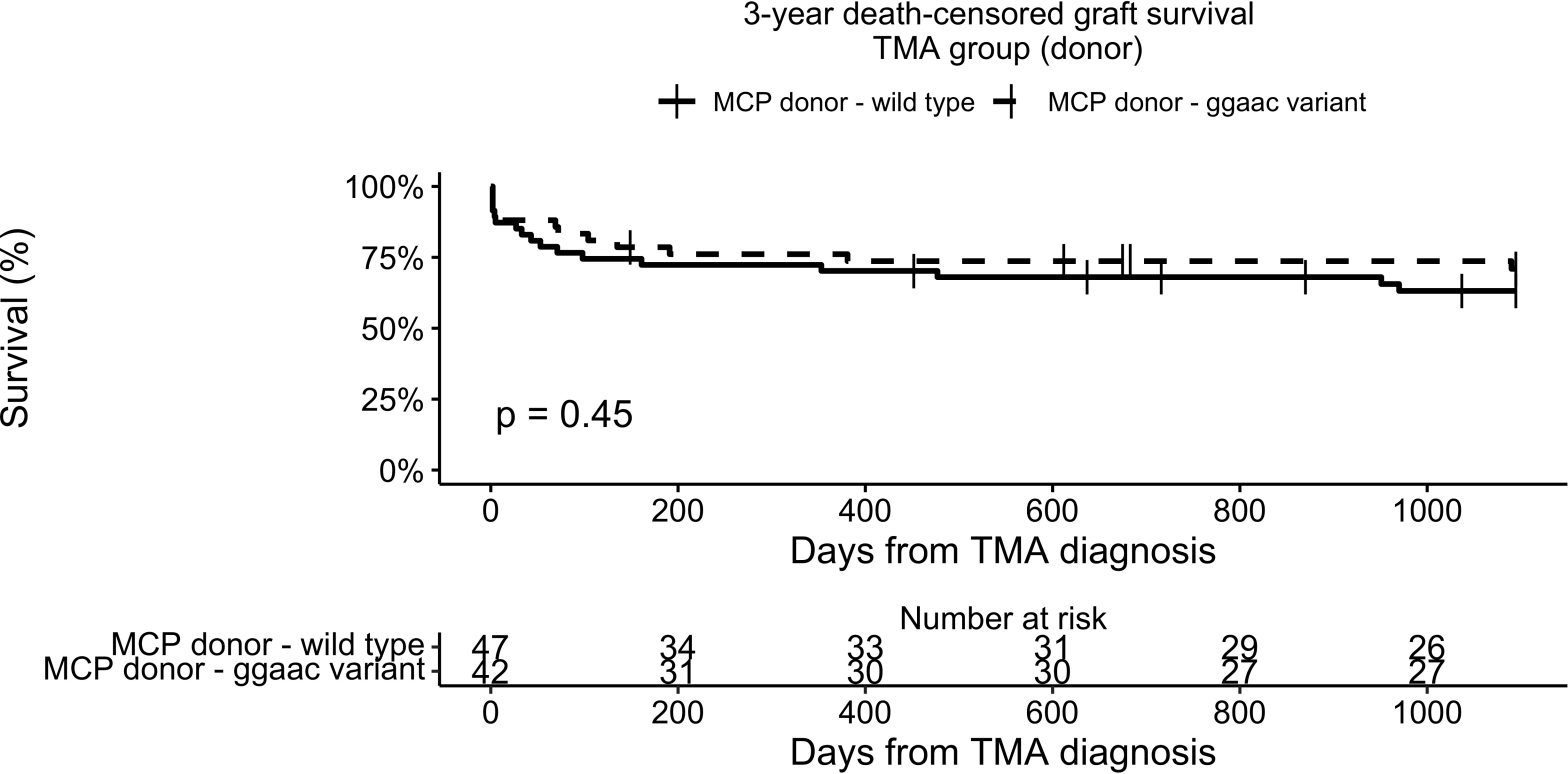
3-year death-censored graft survival in the TMA group according to donor MCP.

**Figure 3:**
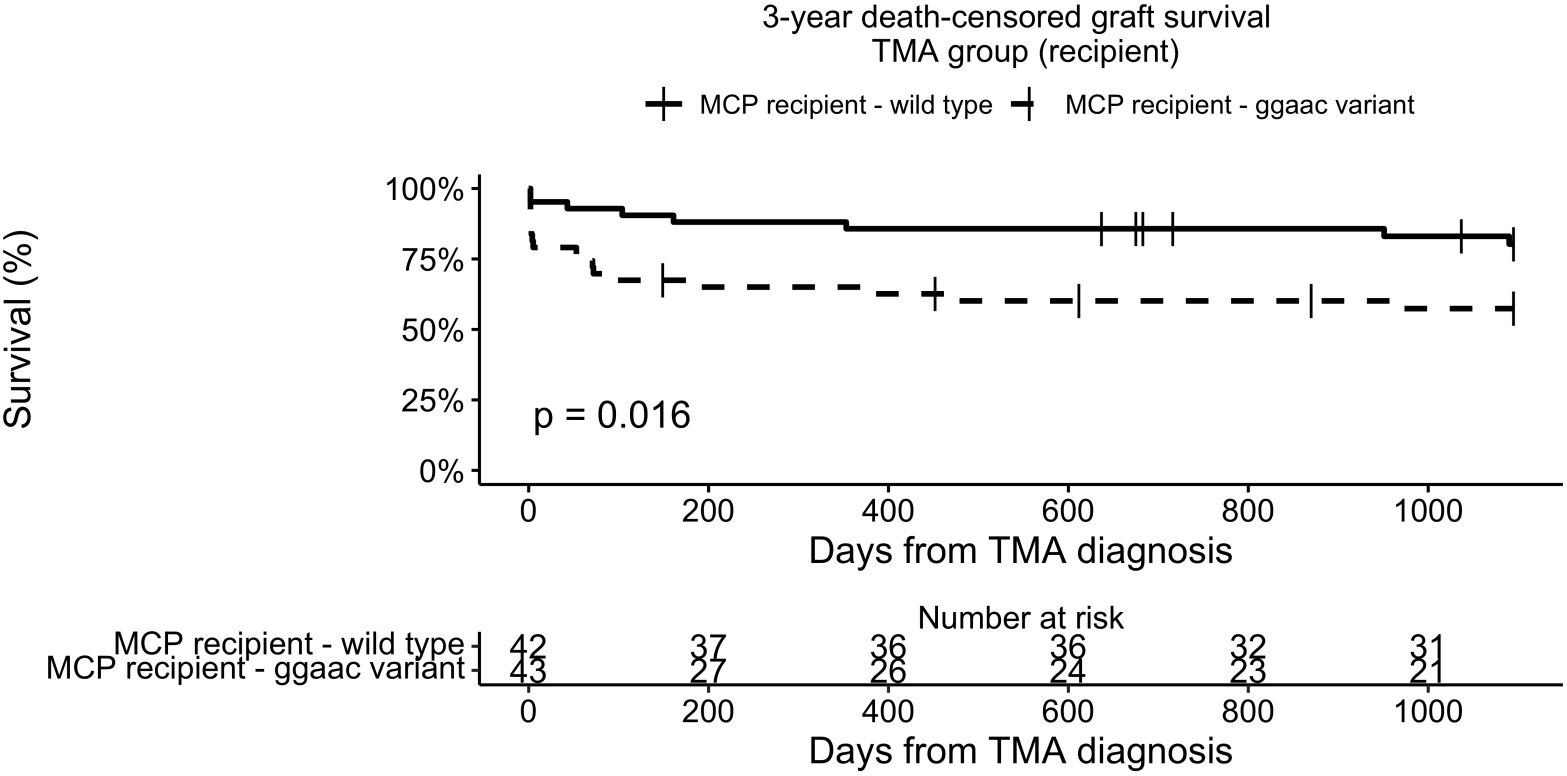
3-year death-censored graft survival in the TMA group according to recipient MCP.

Three-year death-censored graft survival in the TMA group was unaffected by donor *MCPggaac* variant (63.2% in wild-type, 70.9 in *MCPggaac* variant carriers, p = 0.45, **Figure 2**), while it was significantly decreased in association with recipient *MCPggaac* variant (80.3% in wild-type, 57.4% in *MCPggaac* variant carriers, p = 0.016, **Figure 3**).

We have further analyzed clinical factors according to the *MCPggaac* haplotype status in the TMA group. Apart from more frequently observed males and higher peak pretransplant PRA in wild-type group, there were no differences found (**Table 3**). Next, multivariable analysis was performed and recipients’ *MCPggaac* status was found to be independently associated with a decreased 3-year death-censored graft survival in patients with *de novo* TMA (**Table 4**).

**Table 3:** Clinical parameters according to *MCPggaac* haplotype in the TMA group.

Parameter	Wild-type homozygotes (n = 42)	MCPggaac variant carriers (n = 43)	p-value
**Median recipient age, years (IQR)**	48.5 (43 – 55)	57 (45 – 61.5)	0.084
**Female recipient, No. (%)**	11 (26.2)	22 (51.2)	**0.032**
**Median donor age, years (IQR)**	55 (42.3 – 61.5)	54 (46 – 60)	0.909
**Female donor, No. (%)**	24 (57.1)	23 (53.5)	0.904
**Median cold ischemia time, hours (IQR)**	17 (15.2 – 18.8)	16.3 (13.8 – 18.9)	0.474
**Median dialysis vintage duration, month (IQR)**	24 (15 – 50.3)	23 (11.5 – 31.5)	0.167
**Median peak preTx PRA (IQR)**	11 (4.5 – 34)	4 (2 – 17)	**0.041**
**Median HLA mismatch (IQR)**	4 (3 – 4)	3 (2.5 – 4)	0.348
**Retransplantation, No. (%)**	9 (21.4)	5 (11.6)	0.355
**T-cell depletive induction, No. (%)**	23 (54.8)	17 (39.5)	0.234
**No rejection at TMA diagnosis, No. (%)**	29 (69)	28 (65.1)	0.836
**T-cell mediated rejection, No. (%)**	6 (14.3)	5 (11.6)	
**Antibody mediated rejection, No. (%)**	7 (16.7)	9 (20.9)	

TMA, thrombotic microangiopathy; SD, standard deviation; preTx, pretransplantation; PRA, panel-reactive antibodies; HLA, human leukocyte antigen. Bold values denote statistical significance at the p < 0.05 level.

**Table 4:** Factors associated with 3-year death censored graft survival in the TMA group, univariable and multivariable Cox regression.

Parameter	Univariable regression			Multivariable regression		
	HR	95% CI	*p-value*	HR	95% CI	*p-value*
**Recipient age**	1.00	0.96 – 1.03	0.8			
**Female recipient**	2.09	0.97 – 4.53	0.06			
**Donor age**	1.01	0.98 – 1.04	0.4			
**Female donor**	0.77	0.36 – 1.66	0.5			
**Cold ischemia time**	1.25	1.09 – 1.42	**<0.001**	1.20	1.06 – 1.36	**0.003**
**Dialysis vintage duration**	1.01	1.00 – 1.02	0.12			
**Peak preTx PRA**	1.01	1.00 – 1.02	0.073			
**HLA mismatch**	1.03	0.74 – 1.44	0.9			
**Retransplantation**	2.53	1.06 – 6.05	**0.037**	2.22	0.72 – 6.88	0.2
**T-cell depletive induction**	1.73	0.79 – 3.77	0.2			
**TCMR at TMA diagnosis**	1.11	0.32 – 3.88	0.9	1.49	0.41 – 5.44	0.5
**ABMR at TMA diagnosis**	3.44	1.48 – 8.00	**0.004**	2.90	0.95 – 8.85	0.061
***MCPggaac* variant carrier**	2.69	1.17 – 6.19	**0.02**	3.83	1.42 – 10.4	**0.008**

HR, hazard ratio; 95% CI, 95% confidence interval; preTx, pretransplantation; PRA, panel-reactive antibodies; HLA, human leukocyte antigen; TMA, thrombotic microangiopathy; TCMR, T-cell mediated rejection; ABMR, antibody mediated rejection. Bold values denote statistical significance at the p < 0.05 level.

**Figure 4:**
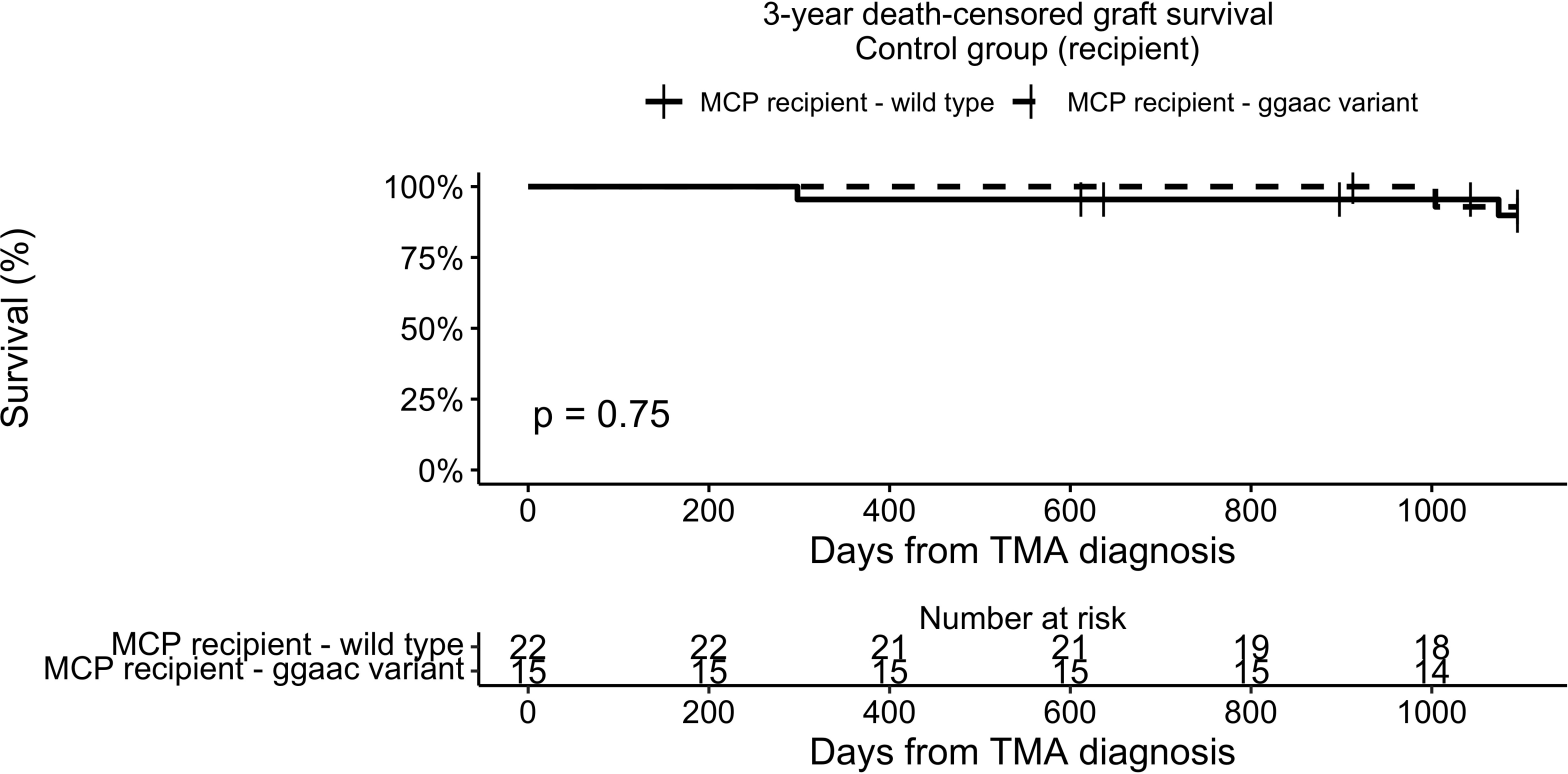
3-year death-censored graft survival in the control group according to recipient MCP.

To analyze the possible interaction between graft survival in TMA-free KTR and *MCPggaac*, we analyzed the presence of *MCPggaac* in the Control group. There was no association between the *MCPggaac* haplotype and graft survival in recipients without TMA (89.8% in wild-type, 92.9% in *MCPggaac* variant carriers, p = 0.75, **Figure 4**).

We also performed analysis of graft survival for heterozygotes and homozygotes separately, low prevalence of homozygotes in both recipients and donors, however, is likely the reason behind the absence of effect of homozygotes on graft survival (**Supplementary Figures 3**–**>5**).

## Discussion

In this study, we demonstrated in kidney transplant recipients with *de novo* TMA that patients carrying the *MCPggaac* haplotype have significantly impaired graft survival compared to recipients carrying the wild-type *MCP* on both chromosomes (membrane cofactor protein, CD46).

It is known that the *MCPggaac* haplotype is overrepresented in patients with atypical hemolytic uremic syndrome, a prototypical complement-driven thrombotic microangiopathy. The *MCPggaac* haplotype is associated with an increased risk and severity of aHUS (7). Furthermore, it has been shown that constituents of the *MCPggaac* haplotype are associated with a decreased gene transcription of MCP *in vitro* (7).

Membrane cofactor protein (MCP), encoded by the *CD46* gene is a membrane bound protein that was originally described as a cofactor for the cleavage of C3b and C4b by complement factor I (8). However, the functions of MCP are far more complicated than initially thought. Apart from the important functions in fertility (9) and host-pathogen interactions (10), it is a vital part of Th1 response activation (11). MCP regulates T-cell metabolic switch, proliferation, polarization towards Th1, and also Th1 response contraction.

Th1 response regulated by MCP is accompanied by significant interferon-γ (IFN-γ) secretion. Jodele et al. showed that TMA after bone marrow transplantation (TA-TMA) has a significant interferon signature and they proposed an interferon loop hypothesis of perpetual endothelial injury in TA-TMA (12). It might be speculated that a decreased MCP expression is associated with a decreased contraction of Th1 response and pathologically prolonged IFN-γ production.

The decreased expression of MCP due to the presence of the *MCPggaac* haplotype was, however, recently disputed by Frimat et al. who studied the expression of MCP on granulocytes and endothelial cells and found no difference between the wild-type and the *MCPggaac* haplotype (13). Even though *MCPggaac* haplotype might not be associated with a significant decrease in MCP expression, the clinical evidence of a higher penetrance and severity of aHUS remains nevertheless strong (7, 14, 15).

In this study we showed that in case of *de novo* TMA in kidney transplantation, recipients carrying the *MCPggaac* haplotype experienced an impaired graft survival more often than patients carrying only the wild-type *CD46* gene. This finding is counterintuitive since MCP, the product of the *CD46* gene, is membrane bound and therefore the cells of the graft origin retain donor MCP. However, in our study the donor *MCP* haplotype did not affect graft survival.

The complex findings of normal tissue expression of MCP, an increased risk of aHUS, and an increased risk of graft loss in *de novo* TMA group might be explained by the association between the *MCPggaac* haplotype and a perturbed expression of other complement regulatory genes within regulators of complement activation gene cluster, as suggested by Esparza-Gordillo and colleagues (7). It has also been suggested that the *MCPggaac* haplotype exerts its effects only in patients with aHUS that carry other complement gene mutations (7, 15). Therefore, it is possible that patients with both *de novo* TMA and the *MCPggaac* haplotype might carry further undiagnosed complement gene variation(s).

The role of one SNP of the *MCPggaac* haplotype (rs2796267) in kidney transplant recipients was studied in 334 kidney transplant recipients by Park et al. (16) They showed that the carriers of this SNP were at a higher risk of acute rejection, but not at a higher risk of graft loss. These results suggest that the *MCPggaac* haplotype exerts its negative effects on graft survival only in patients with other pathogenic hits, such as complement overactivation in the case of *de novo* TMA.

In our previous study that used similar cohort, we have shown that patients with both *de novo* TMA and ABMR present with the worst graft survival. In this study, however, we found that presence of MCPggaac seems to be a stronger predictor of graft survival.

The limitations of this study include retrospective design, small control group and small power to detect modifying effects of other gene variants.

In conclusion, we have found that the recipients’ *MCPggaac* haplotype represents a hereditary modifying factor affecting kidney graft survival in *de novo* TMA. Detection of the *MCPggaa*c haplotype may help to identify patients at risk of premature graft loss in case of *de novo* TMA who may benefit from emerging complement-targeting therapies.

## Data availability statement

The original contributions presented in the study are included in the article/**Supplementary Materials**. Further inquiries can be directed to the corresponding author.

## Ethics statement

The studies involving human participants were reviewed and approved by Ethics Committee of the Institute for Clinical and Experimental Medicine and Thomayer Hospital. Written informed consent for participation was not required for this study in accordance with the national legislation and the institutional requirements.

## Author contributions

VP participated in research design and performance, data acquisition, data analysis, and manuscript writing. DC participated in sample and data analysis. PH participated in sample acquisition and preparation. AS participated in sample and data analysis. MK participated in data analysis. AS participated in sample acquisition and preparation. ZP participated in research design, manuscript writing, sample and data analysis. OV participated in research design and performance, data acquisition, data analysis, and manuscript writing. All authors contributed to the article and approved the submitted version.

## Funding

Supported by the Ministry of Health of the Czech Republic NU22-C-126 and by its conceptual development of research organizations (Institute for Clinical and Experimental Medicine – IKEM, IN 00023001). Supported by the project National Institute for Research of Metabolic and Cardiovascular Diseases (Programme EXCELES, Project No. LX22NPO5104) - Funded by the European Union - Next Generation EU. Project no. TKP2021-EGA-24 (MOLORKIV) has been implemented with the support provided by the Ministry of Innovation and Technology of Hungary from the National Research, Development and Innovation Fund, financed under the TKP2021-EGA funding scheme. DC received research funding from the Premium Postdoctoral Fellowship Program of the Hungarian Academy of Sciences (PPD2018-016/2018).

## Acknowledgments

The authors of this publication are members of the European reference Network for Rare Kidney Diseases (ERKNet).

## Conflict of interest

The authors declare that the research was conducted in the absence of any commercial or financial relationships that could be construed as a potential conflict of interest.

## Publisher’s note

All claims expressed in this article are solely those of the authors and do not necessarily represent those of their affiliated organizations, or those of the publisher, the editors and the reviewers. Any product that may be evaluated in this article, or claim that may be made by its manufacturer, is not guaranteed or endorsed by the publisher.
