# Supplementary material for: MCPggaac haplotype is associated with poor graft survival in kidney transplant recipients with de novo thrombotic microangiopathy
Source: Front Immunol. 2022 Sep 14;13:985766. doi: 10.3389/fimmu.2022.985766 (PMC9519137; doi:10.3389/fimmu.2022.985766)
Supplement: Supplementary file 1 [file DataSheet_1.docx]

**SUPPLEMENTARY MATERIAL**

**Table of Contents:**

**Supplementary Table 1.** Single nucleotide polymorphisms and haplotypes in complement genes analyzed in the study

**Supplementary Table 2.** Selected Banff scores in the TMA diagnosis graft biopsy

**Supplementary Table 3.** Causes of end stage kidney disease

**Supplementary Table 4.** Systemic manifestation of TMA

**Supplementary Table 5.** Maintenance immunosuppression at the TMA diagnosis

**Supplementary Table 6.** Treatment of TMA

**Supplementary Table 7.** Associations between graft loss in 3 years and recipients’ or donors’ SNPs and haplotypes

**Supplementary Figure 1.** 3-year death-censored graft survival comparing TMA group and Control group

**Supplementary Figure 2.** 3-year death-censored graft survival in the TMA group according to the rejection findings

**Supplementary Figure 3.** 3-year death-censored graft survival in the control group in relation to MCPggaac presence in recipients (wild type, heterozygotes, homozygotes)

**Supplementary Figure 4.** 3-year death-censored graft survival in the TMA group in relation to MCPggaac presence in recipients (wild type, heterozygotes, homozygotes)

**Supplementary Figure 5.** 3-year death-censored graft survival in the TMA group in relation to MCPggaac presence in donors (wild type, heterozygotes, homozygotes)

**Supplementary Table 1. Single nucleotide polymorphisms and haplotypes in complement genes analyzed in the study**

| **Gene** | **SNP/haplotype** | **SNP ID** | **Previous data on function and disease associations** | **Studied haplotype(s)** |
| --- | --- | --- | --- | --- |
| ***CFH*** | -257C/T (c.-331C/T) | rs3753394 | Rare allele is a reported risk factor for aHUS (alone or as part of the H3 haplotype) (PMID: 14583443, 15784724) but not for dense deposit disease (PMID: 21784901). | CFH H3 involving  -331T  c.1204C  c.2016C  c.2808T |
|  | Y402H (c.1204C>T) | rs1061170 | Rare allele is a reported risk factor for dense deposit disease (PMID: 16299065, 21784901, 22456601) but not for aHUS (PMID: 14583443, 12960213). Functional studies showed that the H402 variant exhibits a significantly reduced binding to C-reactive protein and heparin (PMID: 17339482, 17360715). |  |
|  | Q672Q (c.2016A>C) | rs3753396 | Rare allele is a reported risk factor for aHUS (alone or as part of the H3 haplotype) (PMID: 14583443, 15784724, 12960213, 22153652) but not for dense deposit disease (PMID: 16299065, 21784901). |  |
|  | E936D (c.2808G>T) | rs1065489 | Rare allele is a reported risk factor for aHUS (alone or as part of the H3 haplotype) (PMID: 14583443, 15784724, 12960213, 22153652) but not for dense deposit disease (PMID: 16299065, 21784901). |  |
| ***CD46*** | -547A/G (c.-652A/G) | rs2796267 | Carriage of rs2796267 in the recipient is associated with higher risk of acute rejection after kidney transplant (PMID: 27234742)  Carriage of rs2796267 and rs2796268 in the donor is associated with higher risk of acute rejection after kidney transplant (PMID: 29867953)  rs2796267 and rs2796268: the construct containing the rare alleles of these polymorphisms showed a 25% lower transcriptional activity than the wild type in vitro, suggesting the lower expression of MCP on the cell surface (PMID: 15661753).  rs2796267, rs2796268 and rs1962149: Rare alleles of these polymorphisms and their haplotype referred as MCPggaac is a risk factor for developing aHUS (PMID: 15661753, 15784724). | MCPggaac involving  c.-652G  c.-366G  c.989-78A |
|  | -261A/G (c.-366A/G ) | rs2796268 |  |  |
|  | IVS9-78G/A (c.989-78G>A) | rs1962149 |  |  |
| ***C3*** | R102G (c.304C>G) | rs2230199 | Rare allele is a reported susceptibility factor for dense deposit disease (PMID: 21784901).  In hemolysis assays G102 activated the alternative pathway more efficiently and had higher hemolytic activity compared with the R102 C3 protein as the affected amino acid change influences the efficiency of regulation by factor H (PMID: 21555552) |  |
|  | P314L (c.941C>T) | rs1047286 | Rare allele is a reported susceptibility factor for dense deposit disease (PMID: 21784901).  Carriers of the rare allele were reported to have lower alternative pathway activity (AP50) than those with homozygous wild genotypes (PMID: 21784901). |  |
| ***CFB*** | R32W (c.94C>T) | rs12614 | In vitro functional studies showed that the W32 containing protein has a decreased binding ability to C3b (40-50% weaker than the wild-type protein) and reduced C3 deposition was observed on endothelial cells in the presence of the variant protein (PMID: 24652797, 19255449). | Haplotypes  containing either  c.94T or  c.95A |
|  | R32Q (c.95G>A) | rs641153 | In vitro functional studies showed that the Q32 containing protein has a decreased binding ability to C3b (40-50% weaker than the wild-type protein) and reduced C3 deposition was observed on endothelial cells in the presence of the variant protein (PMID: 24652797).  The 32Q containing factor B protein has decreased potential to form convertase and amplify complement activation (PMID: 19255449, 16518403). |  |

**Supplementary Table 2. Selected Banff scores in the TMA diagnosis graft biopsy.**

| **Patient Nr.** | **C4d** | **g score** | **cg score** | **mm score** | **i score** | **t score** | **IFTA score** | **ptc score** | **v score** | **cv score** | **ah score** |
| --- | --- | --- | --- | --- | --- | --- | --- | --- | --- | --- | --- |
| **1** | 3 | 2 | 2 | 3 | 1 | 1 | 0 | 2 | 1 | 1 | 3 |
| **2** | 2 | 3 | 1 | 0 | 0 | 1 | 0 | 0 | 0 | 0 | 1 |
| **3** | 0 | 3 | 2 | 2 | 2 | 3 | 3 | 0 | 1 | 1 | 0 |
| **4** | 0 | 1 | 0 | 0 | 1 | 1 | 0 | 0 | 0 | 3 | 1 |
| **5** | 0 | 0 | 0 | 0 | 0 | 0 | 1 | 1 | 1 | 1 | 2 |
| **6** | 0 | 0 | 0 | 0 | 0 | 0 | 0 | 0 | 0 | 1 | 3 |
| **7** | 0 | 0 | 0 | 0 | 0 | 0 | 1 | 1 | 0 | 1 | 2 |
| **8** | 0 | 1 | 0 | 0 | 0 | 0 | 1 | 2 | 0 | 1 | 1 |
| **9** | 2 | 0 | 0 | 0 | 0 | 0 | 1 | 0 | 0 | 0 | 1 |
| **10** | 0 | 0 | 0 | 0 | 0 | 0 | 2 | 0 |  | 0 | 1 |
| **11** | 0 | 0 | 0 | 0 | 0 | 0 | 0 | 0 | 0 | 1 | 2 |
| **12** | 0 | 1 | 0 | 0 | 1 | 1 | 0 | 0 | 0 | 1 | 3 |
| **13** | 0 |  |  |  | 0 | 1 | 0 | 0 | 0 | 1 | 2 |
| **14** | 0 | 1 | 0 | 0 | 2 | 1 | 0 | 0 | 2 | 1 | 1 |
| **15** | 0 | 0 | 0 | 0 | 0 | 0 | 0 | 0 | 0 | 1 | 1 |
| **16** | 0 | 1 | 2 | 0 | 0 | 1 |  | 0 | 0 | 3 | 2 |
| **17** | 0 | 1 | 0 | 0 | 0 | 0 | 1 | 0 | 0 | 1 | 1 |
| **18** | 0 | 1 | 0 | 0 | 0 | 0 | 1 | 0 | 0 | 1 | 2 |
| **19** | 0 | 0 | 0 | 0 | 0 | 0 | 1 | 0 | 3 | 2 | 3 |
| **20** | 0 | 0 | 0 | 0 | 0 | 0 | 0 |  | 3 | 0 | 0 |
| **21** | 1 | 0 | 0 | 0 | 0 | 0 | 1 | 0 | 0 | 3 | 3 |
| **22** | 0 | 0 | 0 | 0 | 0 | 0 | 0 |  |  | 2 | 3 |
| **23** | 1 | 0 | 0 | 0 | 0 | 0 | 1 |  | 1 | 1 | 1 |
| **24** | 0 | 0 | 0 | 0 | 0 | 0 | 1 | 0 | 0 | 2 | 1 |
| **25** | 0 | 2 | 0 |  |  | 3 | 1 | 1 | 1 | 2 | 2 |
| **26** | 0 |  |  |  | 0 | 0 | 1 | 0 | 0 | 2 | 2 |
| **27** | 3 | 0 | 0 | 0 | 0 | 0 | 1 | 0 | 0 | 3 | 2 |
| **28** | 1 | 0 | 0 | 0 | 0 | 0 | 1 | 0 | 0 | 3 | 3 |
| **29** | 0 | 0 | 0 | 0 | 0 | 0 | 1 | 0 | 0 | 1 | 1 |
| **30** | 3 | 2 | 0 | 0 | 0 | 0 | 0 | 0 | 0 | 0 | 0 |
| **31** | 0 | 2 | 0 | 0 | 0 | 0 | 1 | 0 | 0 | 1 |  |
| **32** | 0 | 0 | 0 | 0 | 1 | 2 | 0 | 0 | 0 | 3 | 3 |
| **33** | 3 | 1 | 0 | 0 | 0 | 1 | 0 | 1 | 0 | 0 | 0 |
| **34** | 0 | 0 | 0 | 0 | 0 | 0 | 1 | 0 | 0 | 3 | 1 |
| **35** | 0 | 3 | 0 | 0 | 2 | 1 |  | 0 | 1 | 0 | 0 |
| **36** | 0 | 0 | 2 | 1 | 1 | 0 | 2 | 0 | 0 | 1 | 2 |
| **37** | 0 | 0 | 0 | 0 | 1 | 1 | 0 | 0 | 1 | 0 | 0 |
| **38** | 0 | 0 | 0 | 0 | 0 | 0 | 2 | 0 | 0 | 1 | 3 |
| **39** | 0 | 0 | 0 | 1 | 0 | 1 | 0 | 0 | 0 | 3 | 2 |
| **40** | 3 | 3 | 3 | 0 | 1 | 1 | 0 | 1 | 3 | 2 | 2 |
| **41** | 0 | 0 | 1 | 0 | 0 | 0 | 0 | 0 | 2 | 0 | 3 |
| **42** | 0 | 0 | 0 | 0 | 0 | 1 | 0 | 0 | 0 | 2 | 1 |
| **43** | 0 | 0 | 0 | 0 | 0 | 2 | 0 | 0 | 0 | 1 | 1 |
| **44** | 0 | 0 | 0 | 0 | 0 | 0 | 0 | 0 | 0 | 0 | 0 |
| **45** | 0 | 0 | 0 | 0 | 0 | 1 | 0 | 0 | 0 | 1 | 1 |
| **46** | 0 | 0 | 0 | 0 | 0 | 0 | 0 | 0 | 0 | 1 | 1 |
| **47** | 0 | 1 | 0 | 0 | 0 | 0 | 0 | 0 | 0 | 1 | 1 |
| **48** | 0 | 0 | 0 | 0 | 0 | 0 | 0 | 0 | 0 | 2 | 1 |
| **49** | 0 | 2 | 1 | 0 | 0 | 0 | 0 | 1 |  |  | 1 |
| **50** | 0 | 0 | 0 | 0 | 0 | 0 | 1 | 0 |  | 2 | 2 |
| **51** | 0 | 0 | 0 | 0 | 0 | 0 | 1 | 0 | 0 | 2 | 2 |
| **52** | 1 | 2 | 0 | 0 | 0 | 0 | 2 | 0 | 0 | 3 | 3 |
| **53** | 0 | 1 | 0 | 0 | 0 | 0 | 1 | 0 | 0 | 0 | 1 |
| **54** | 0 |  |  |  | 0 | 0 | 1 | 0 | 0 | 2 |  |
| **55** | 0 | 0 | 0 | 0 | 0 | 0 | 1 | 0 | 0 | 2 | 1 |
| **56** | 0 | 0 | 0 | 0 | 1 | 2 | 1 | 0 | 0 | 2 | 0 |
| **57** | 0 | 0 | 0 | 0 | 0 | 0 | 0 | 0 | 0 | 2 | 3 |
| **58** | 0 | 0 | 0 | 0 | 0 | 0 | 1 | 0 | 0 | 2 | 3 |
| **59** | 0 | 1 | 0 | 0 | 0 | 0 | 1 | 0 | 0 | 3 | 2 |
| **60** | 3 | 3 | 0 | 0 | 0 | 1 | 1 | 2 | 0 | 2 | 1 |
| **61** | 0 | 3 | 1 | 1 | 1 | 1 | 1 | 2 | 1 | 1 | 2 |
| **62** | 0 | 0 | 0 | 0 | 0 | 1 | 0 | 0 | 0 | 1 | 1 |
| **63** | 0 | 0 | 0 | 0 | 0 | 1 | 0 | 0 | 0 | 1 | 1 |
| **64** | 3 | 2 | 0 | 0 | 0 | 0 | 0 | 0 | 0 | 1 | 1 |
| **65** | 0 |  |  |  |  |  | 1 |  |  |  | 1 |
| **66** | 0 | 0 | 0 | 0 | 1 | 2 | 1 | 0 | 0 | 1 | 2 |
| **67** | 0 | 0 | 0 | 0 | 0 | 0 | 1 | 0 | 0 | 3 | 3 |
| **68** | 3 | 2 | 0 | 0 | 0 | 0 | 0 | 0 | 0 | 2 | 1 |
| **69** | 0 | 0 | 0 | 0 | 0 | 0 | 1 | 0 | 0 | 1 | 1 |
| **70** | 0 | 0 | 0 | 0 | 0 | 0 | 1 | 0 | 3 | 3 | 1 |
| **71** | 0 | 1 | 0 | 0 | 0 | 0 | 1 | 0 | 0 | 0 | 1 |
| **72** | 3 | 0 | 0 | 0 | 0 | 0 | 0 | 0 | 0 | 3 | 2 |
| **73** | 0 | 0 | 0 | 1 | 0 | 0 | 0 | 0 | 0 | 0 | 0 |
| **74** | 3 | 2 | 0 | 0 | 1 | 1 | 0 | 3 | 1 | 1 | 1 |
| **75** | 0 | 0 | 0 | 0 | 0 | 0 | 0 | 0 | 0 | 3 | 2 |
| **76** | 0 | 1 | 0 | 0 | 0 | 0 | 1 | 0 | 0 | 1 | 1 |
| **77** | 0 | 0 | 0 | 0 | 0 | 0 | 0 | 0 | 0 | 0 | 1 |
| **78** | 0 | 0 | 0 | 0 | 0 | 0 | 0 | 0 | 0 | 0 | 0 |
| **79** | 0 | 1 | 0 | 0 | 0 | 1 | 1 | 0 | 0 | 2 | 2 |
| **80** | 1 | 0 | 0 | 0 | 0 | 0 | 0 | 0 | 0 | 1 | 3 |
| **81** | 0 | 0 | 0 | 0 | 0 | 0 | 0 | 0 | 0 | 1 | 1 |
| **82** | 1 | 0 | 0 | 0 | 0 | 0 | 1 | 0 | 0 | 1 | 1 |
| **83** | 1 | 2 | 3 | 0 | 0 | 0 | 1 | 2 | 1 | 3 | 3 |
| **84** | 3 | 0 | 0 | 0 | 0 | 0 | 2 | 0 | 0 | 3 | 2 |
| **85** | 1 | 0 | 0 | 0 | 0 | 0 | 1 | 0 | 0 | 1 | 1 |
| **86** | 0 | 2 | 0 | 0 | 1 | 3 | 1 | 2 | 2 | 1 | 2 |
| **87** | 0 | 0 | 0 | 0 | 0 | 0 | 1 | 0 | 0 | 1 | 1 |
| **88** | 2 | 0 | 0 | 3 | 1 | 1 | 3 |  | 0 | 2 | 3 |
| **89** | 0 | 2 | 0 | 0 | 0 | 0 | 0 | 0 | 0 | 1 | 1 |
| **90** | 0 | 0 | 1 | 0 | 0 | 0 | 0 |  | 0 | 1 | 2 |

**Abbreviations:** g, glomerulitis; cg, transplant glomerulopathy; mm, mesangial matrix expansion; i, interstitial inflammation; t, tubulitis; IFTA, interstitial fibrosis/tubular atrophy; ptc, peritubular capilaritis; v, intimal arteritis; cv, arterial intimal fibrosis; ah, arteriolar hyalinosis.

**Supplementary Table 3. Causes of end stage kidney disease**

| **Underlying nephropathy** | **n (%)** |
| --- | --- |
| **ANCA associated vasculitis** | 5 (5.6%) |
| **Polycystic kidney disease** | 13 (14.4%) |
| **Glomerulonephritis otherwise unspecified** | 9 (10%) |
| **Diabetic kidney disease** | 10 (11.1%) |
| **Hereditary nephropathy** | 7 (7.8%) |
| **IgA nephropathy** | 10 (11.1%) |
| **Membranous nephropathy** | 2 (2.2%) |
| **Membranoproliferative glomerulonephritis** | 3 (3.3%) |
| **Reflux nephropathy** | 6 (6.7%) |
| **Tubulointerstitial nephritis** | 10 (11.1%) |
| **Unknown** | 4 (4.4%) |
| **Vascular/Hypertensive nephropathy** | 11 (12.2%) |

**Abbreviations**: ANCA, anti-neutrophil cytoplasm antibody; IgA, immunoglobulin A.

**Supplementary Table 4. Systemic manifestation of TMA**

| **Systemic manifestation** | **n (%)** |
| --- | --- |
| **Lactate dehydrogenase (1.5x ULN)** | 71 (56.3%) |
| **Anemia (Hb < 11 g/dL)** | 79 (87.8%) |
| **Thrombocytopenia (PLT < 150 000/μL)** | 59 (65.6%) |
| **Schistocytes (>1%)** | 28 (41.8%) |

**Abbreviations:** ULN, upper limit of normal; Hb, hemoglobin; PLT, platelets.

**Supplementary Table 5. Maintenance immunosuppression at the TMA diagnosis**

| **Nr** | **Immunosuppression** |
| --- | --- |
| **4** | Cyclosporin A + MMF + steroids |
| **5** | Cyclosporin A + MMF + steroids |
| **6** | Cyclosporin A + MMF + steroids |
| **16** | Cyclosporin A + MMF + steroids |
| **17** | Cyclosporin A + MMF + steroids |
| **70** | Cyclosporin A + MMF + steroids |
| **82** | Cyclosporin A + MMF + steroids |
| **50** | Cyclosporin A + mTORi + steroids |
| **71** | mTORi + MMF |
| **3** | mTORi + steroids |
| **38** | mTORi + steroids |
| **1** | Tacrolimus + MMF + steroids |
| **2** | Tacrolimus + MMF + steroids |
| **7** | Tacrolimus + MMF + steroids |
| **8** | Tacrolimus + MMF + steroids |
| **9** | Tacrolimus + MMF + steroids |
| **10** | Tacrolimus + MMF + steroids |
| **11** | Tacrolimus + MMF + steroids |
| **13** | Tacrolimus + MMF + steroids |
| **14** | Tacrolimus + MMF + steroids |
| **15** | Tacrolimus + MMF + steroids |
| **18** | Tacrolimus + MMF + steroids |
| **19** | Tacrolimus + MMF + steroids |
| **20** | Tacrolimus + MMF + steroids |
| **21** | Tacrolimus + MMF + steroids |
| **22** | Tacrolimus + MMF + steroids |
| **23** | Tacrolimus + MMF + steroids |
| **24** | Tacrolimus + MMF + steroids |
| **25** | Tacrolimus + MMF + steroids |
| **26** | Tacrolimus + MMF + steroids |
| **27** | Tacrolimus + MMF + steroids |
| **28** | Tacrolimus + MMF + steroids |
| **29** | Tacrolimus + MMF + steroids |
| **30** | Tacrolimus + MMF + steroids |
| **31** | Tacrolimus + MMF + steroids |
| **33** | Tacrolimus + MMF + steroids |
| **34** | Tacrolimus + MMF + steroids |
| **35** | Tacrolimus + MMF + steroids |
| **36** | Tacrolimus + MMF + steroids |
| **37** | Tacrolimus + MMF + steroids |
| **39** | Tacrolimus + MMF + steroids |
| **40** | Tacrolimus + MMF + steroids |
| **41** | Tacrolimus + MMF + steroids |
| **42** | Tacrolimus + MMF + steroids |
| **43** | Tacrolimus + MMF + steroids |
| **44** | Tacrolimus + MMF + steroids |
| **46** | Tacrolimus + MMF + steroids |
| **47** | Tacrolimus + MMF + steroids |
| **48** | Tacrolimus + MMF + steroids |
| **52** | Tacrolimus + MMF + steroids |
| **53** | Tacrolimus + MMF + steroids |
| **54** | Tacrolimus + MMF + steroids |
| **55** | Tacrolimus + MMF + steroids |
| **56** | Tacrolimus + MMF + steroids |
| **58** | Tacrolimus + MMF + steroids |
| **59** | Tacrolimus + MMF + steroids |
| **62** | Tacrolimus + MMF + steroids |
| **64** | Tacrolimus + MMF + steroids |
| **65** | Tacrolimus + MMF + steroids |
| **66** | Tacrolimus + MMF + steroids |
| **67** | Tacrolimus + MMF + steroids |
| **68** | Tacrolimus + MMF + steroids |
| **69** | Tacrolimus + MMF + steroids |
| **72** | Tacrolimus + MMF + steroids |
| **73** | Tacrolimus + MMF + steroids |
| **75** | Tacrolimus + MMF + steroids |
| **76** | Tacrolimus + MMF + steroids |
| **77** | Tacrolimus + MMF + steroids |
| **78** | Tacrolimus + MMF + steroids |
| **79** | Tacrolimus + MMF + steroids |
| **80** | Tacrolimus + MMF + steroids |
| **81** | Tacrolimus + MMF + steroids |
| **83** | Tacrolimus + MMF + steroids |
| **84** | Tacrolimus + MMF + steroids |
| **86** | Tacrolimus + MMF + steroids |
| **87** | Tacrolimus + MMF + steroids |
| **88** | Tacrolimus + MMF + steroids |
| **89** | Tacrolimus + MMF + steroids |
| **90** | Tacrolimus + MMF + steroids |
| **12** | Tacrolimus + MMF |
| **45** | Tacrolimus + MMF |
| **60** | Tacrolimus + MMF |
| **51** | Tacrolimus + mTORi + steroids |
| **63** | Tacrolimus + mTORi + steroids |
| **74** | Tacrolimus + mTORi + steroids |
| **32** | Tacrolimus + steroids |
| **49** | Tacrolimus + steroids |
| **57** | Tacrolimus + steroids |
| **85** | Tacrolimus + steroids |
| **61** | Tacrolimus |

**Abbreviations**: mTORi, inhibitor of mechanistic target of rapamycin; MMF, mycophenolate.

**Supplementary Table 6. Treatment of TMA**

| **Treatment** | **n (%)** |
| --- | --- |
| **Calcineurin inhibitor discontinuation/decrease** | 51 (56.7%) |
| **Switch from CNI to mTORi** | 17 (18.9%) |
| **Intravenous immunoglobulin** | 18 (20%) |
| **Anti-CD20** | 6 (6.7%) |
| **Plasma exchange/Plasmapheresis** | 53 (58.9%) |
| **Bortezomib** | 3 (3.3%) |
| **Eculizumab** | 5 (5.6%) |
| **Anti-thymocyte globuline** | 15 (16.7%) |
| **Belatacept** | 6 (6.7%) |

**Abbreviations:** TMA, thrombotic microangiopathy; CNI, calcineurin inhibitor; mTORi, inhibitor of mechanistic target of rapamycin.

**Supplementary Table 7. Associations between graft loss in 3 years and recipients’ or donors’ SNPs and haplotypes**

| **Gene** | **SNP/haplotype (Reference SNP cluster ID)** | **Graft loss in 3years (recipients’ haplotypes and SNPs)** | | | | **Graft loss in 3years (donors’ haplotypes and SNPs)** | | | |
| --- | --- | --- | --- | --- | --- | --- | --- | --- | --- |
|  |  | **WT HM, n (%)** | **HT, n (%)** | **Variant HM, n (%)** | **p-value** | **WT HM, n (%)** | **HT, n (%)** | **Variant HM, n (%)** | **p-value** |
| ***CFH*** | -257C/T (rs3753394) | 12 (30) | 9 (25.7) | 3 (42.9) | 0.654 | 18 (39.1) | 9 (22) | 1 (50) | 0.193 |
|  | Y402H (rs1061170) | 10 (34.5) | 11 (28.9) | 5 (29.4) | 0.878 | 13 (39.4) | 10 (25) | 6 (35.3) | 0.405 |
|  | Q672Q (rs3753396) | 21 (35.6) | 5 (21.7) | 1 (33.3) | 0.480 | 21 (35) | 6 (23.1) | 1 (33.3) | 0.549 |
|  | E936D (rs1065489) | 21 (36.2) | 4 (17.4) | 1 (33.3) | 0.255 | 23 (36.5) | 4 (17.4) | 2 (50) | 0.180 |
|  | CFH H3 haplotype | 21 (33.3) | 4 (22.2) | 1 (33.3) | 0.803 | 25 (38.5) | 3 (12.5) | 1 (100) | **0.009** |
| ***CD46*** | c.-652A/G (rs2796267) | 7 (18.9) | 16 (43.2) | 3 (27.3) | 0.074 | 12 (32.4) | 13 (32.5) | 4 (33.3) | 0.998 |
|  | c.-366A/G (rs2796268) | 7 (23.3) | 17 (37) | 2 (22.2) | 0.383 | 15 (37.5) | 13 (35.1) | 1 (8.3) | 0.152 |
|  | IVS9-78G/A (rs1962149) | 7 (22.6) | 18 (39.1) | 1 (14.3) | 0.186 | 16 (38.1) | 11 (32.4) | 1 (7.7) | 0.118 |
|  | *MCPggaac* haplotype | 8 (19) | 17 (45.9) | 1 (16.7) | **0.026** | 17 (36.2) | 11 (33.3) | 1 (11.1) | 0.337 |
| ***C3*** | R102G (rs2230199) | 16 (27.6) | 10 (40) | 0 (0) | 0.425 | 23 (35.4) | 5 (25) | 1 (20) | 0.572 |
|  | P314L (rs1047286) | 17 (29.3) | 9 (36) | 0 (0) | 0.664 | 22 (34.4) | 6 (27.3) | 1 (25) | 0.787 |
| ***CFB*** | R32W (rs12614) | 21 (32.3) | 5 (27.8) | 0 (0) | 0.745 | 24 (32.9) | 4 (25) | 1 (100) | 0.287 |
|  | R32Q (rs641153) | 21 (31.8) | 5 (29.4) | 0 (0) | 0.783 | 27 (32.5) | 1 (16.7) | 1 (100) | 0.250 |
|  | CFB R32W/Q haplotypes | 16 (32.7) | 10 (32.3) | 0 (0) | 0.390 | 22 (33.3) | 5 (22.7) | 2 (100) | 0.076 |

**Abbreviations**: SNP, single nucleotide polymorphism; CFH, complement factor H; CD46, cluster of differentiation 46; C3, complement factor 3; CFB, complement factor B; WT HM, wild-type homozygote; HT, heterozygote; SNP HM, single nucleotide polymorphisms homozygote; TMA, thrombotic microangiopathy.

**
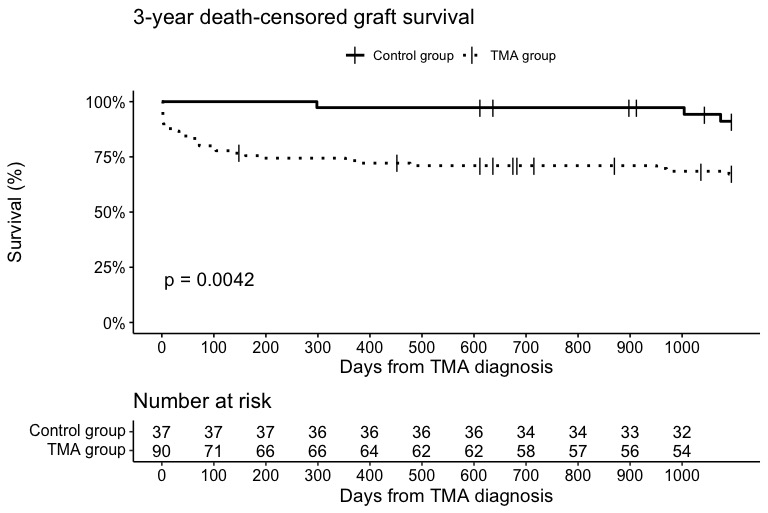
**

**Supplementary Figure 1. 3-year death-censored graft survival comparing TMA group and Control group**

**
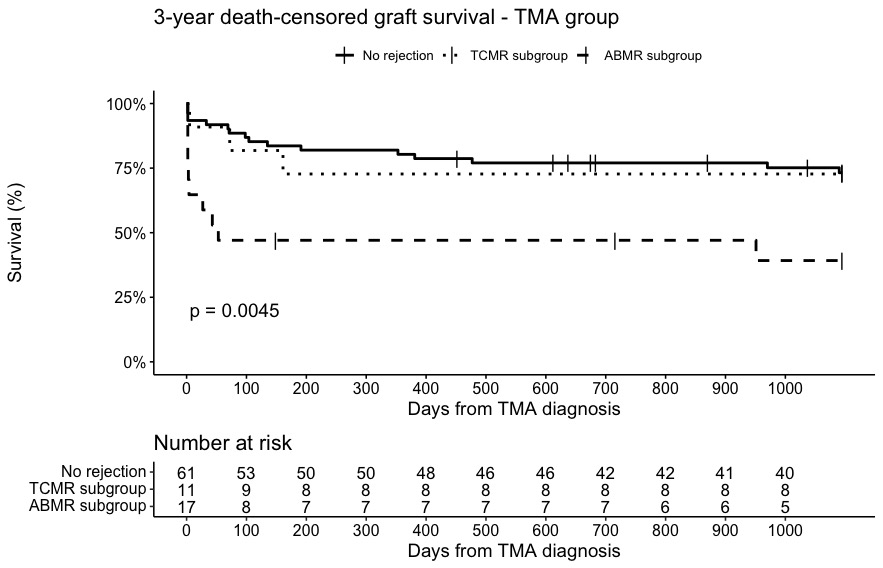
**

**Supplementary Figure 2. 3-year death-censored graft survival in the TMA group according to the rejection findings**

**Supplementary Figure 3. 3-year death-censored graft survival in the control group in relation to MCPggaac presence in recipients (wild type, heterozygotes, homozygotes)**

**Supplementary Figure 4. 3-year death-censored graft survival in the TMA group in relation to MCPggaac presence in recipients (wild type, heterozygotes, homozygotes)**

**Supplementary Figure 5. 3-year death-censored graft survival in the TMA group in relation to MCPggaac presence in donors (wild type, heterozygotes, homozygotes)**
